# Surgical approach to multifocal hepatocellular carcinoma with portal vein thrombosis and arterioportal shunt leading to portal hypertension and bleeding: a case report

**DOI:** 10.1186/1477-7819-10-34

**Published:** 2012-02-13

**Authors:** Francesca Ratti, Federica Cipriani, Michele Paganelli, Gianfranco Ferla, Luca AM Aldrighetti

**Affiliations:** 1 Department of Surgery - Hepatobiliary Surgery Unit; Vita-Salute S.Raffaele University, Milan, Italy

**Keywords:** HCC, portal vein thrombosis, arterioportal shunt, liver surgery

## Abstract

It is reported the case of a 69 years man who presented to the Emergency Room because of pain and abdominal distension from ascites. After admission and paracentesis placement, he developed a digestive hemorrhage due to oesophageal varices from portal ipertension secondary to the formation of a portal shunt concomitant with a multifocal HepatoCellular Carcinoma (HCC) with portal vein thrombosis (PVT). The patient underwent endoscopic varices ligation, twice transarterial embolization (TAE) of arterial branches feeding the shunt and subsequent left hepatectomy. During the postoperative course he developed mild and transient signs of liver failure and was discharged in postoperative day 16. He is alive and disease free 8 months after surgery.

## Background

The aggressiveness and invasiveness of Hepatocellular Carcinoma (HCC) towards the main portal vein and portal vein branches frequently causes the formation of abnormal communications between vessels originating from the hepatic artery and portal vein, creating an arterioportal shunt (APS) [1]. Hemodynamic changes resulting from the formation of this vascular abnormality tend to cause or worsen portal hypertension and therefore to increase the incidence of major complications [2]. Transient complications of portal hypertension lead to overestimation of patient's class of risk, especially regarding postoperative liver failure: their recognition and treatment allows avoidance of inadvertent exclusion from surgery [3], improving long term outcome [4].

It is reported the case of a patient with multifocal HCC complicated by the presence of APS and portal vein thrombosis (PVT) diagnosed after an episode of digestive hemorrhage from oesophageal varices.

## Case presentation

### Patient characteristics and treatment

**Figure 1:**
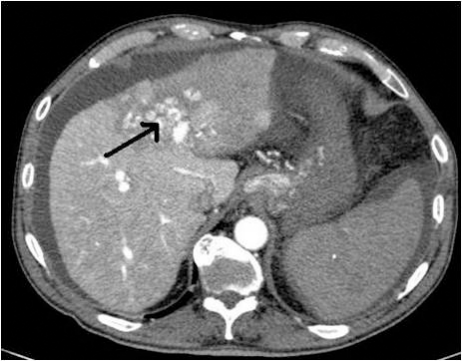
**Confluent HCC lesions in the left lobe of liver**.

**Figure 2:**
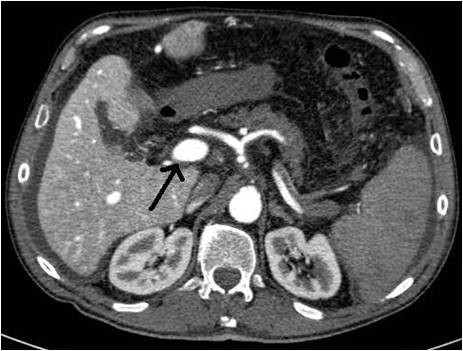
**Hyper-enhanced portal vein during the arterial phase**.

**Figure 3:**
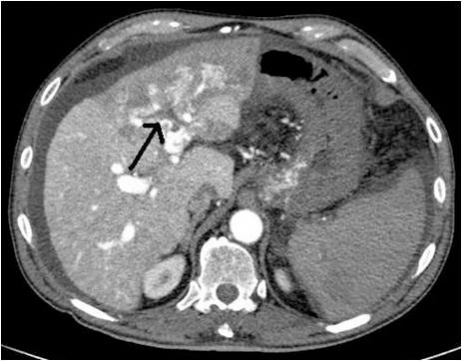
**Tumor thrombus in the left portal vein**.

**Figure 4:**
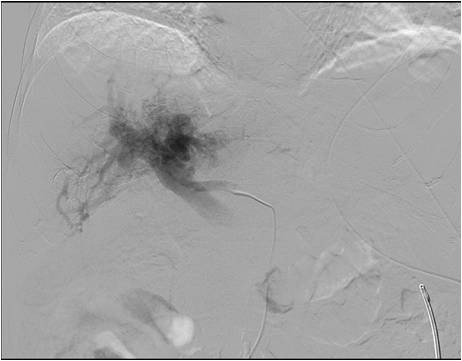
**Preliminary angiography confirming APS presence**.

**Figure 5:**
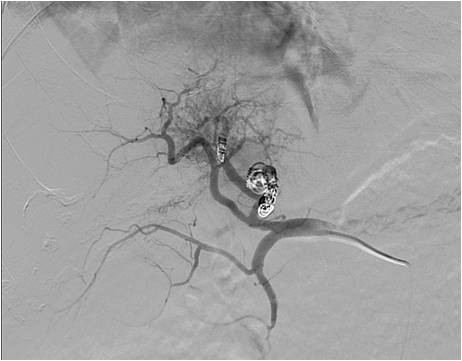
**APS embolization**.

**Figure 6:**
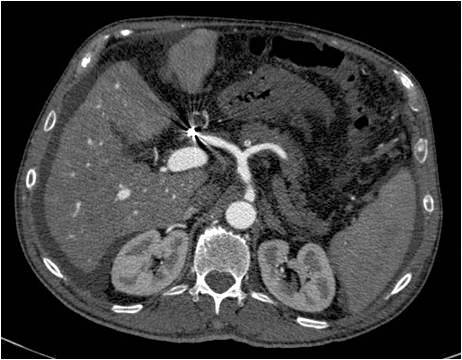
**Persistent contrast enhancement of the portal vein**.

**Figure 7:**
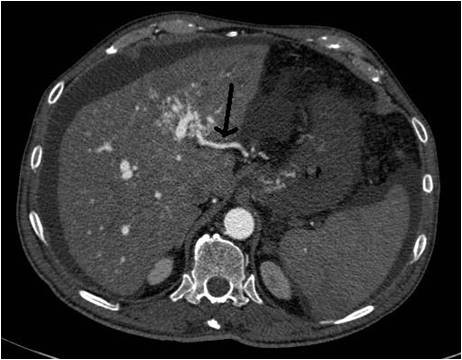
**APS refurnished from artery originating from the left gastric artery**.

A 69-years old male known for post alcoholic liver cirrhosis was admitted at the Division of Internal Medicine through the Emergency Room because of abdominal pain and distension, diarrhea and oliguria. Physical examination revealed abdominal distension because of severe ascites. Murphy and Blumberg signs were not evocable. Laboratory data showed: white blood cells 5.3 10^9/L; haemoglobin 12.6 g/dL; platelets 98 10^9/L; AST/ALT 66/90 U/L; PT ratio 1.10; aPTT ratio 0.92; total bilirubin 0.75 mg/dL; Albumin 41.8 g/L; α-Fetoprotein 249.6 ng/mL; Creatinine 1.06 mg/dL. Child-Pugh class was A7 (the patient took 2 points for ascites). An abdominal ultrasonography revealed severe ascites, multiple lesions in the left liver and absence of blood flow in the left portal vein branch. A paracentesis was then placed with drainage of 3 liters of clear ascites. The day following admission hematemesis and melena occurred due to bleeding from oesophageal varices: the patient underwent upper gastrointestinal endoscopy which revealed III grade blue varices, with red signs and with blood clots in the gastric fundus. Endoscopic band ligation of oesophageal varices was performed with success. Abdominal dynamic computed tomography (CT) revealed low density confluent lesions in all phases in the left lobe of the liver (Figure 1), an hyper-enhanced portal vein during the arterial phase (Figure 2) and a tumor thrombus in the left portal vein causing a filling defect (Figure 3), ascites and splenomegaly. The patient was diagnosed with HCC with severe intratumoral APS, which caused portal hypertension that lead to oesophagogastric varices and hypersplenism. Since the main cause of the portal hypertension was the APS, transcatheter arterial embolization (TAE) was performed twice. The preliminary angiography confirmed the presence of APS (Figure 4) and during the first TAE, branches coming from left hepatic artery were embolized (Figure 5). When a control CT scan was performed, it revealed a persistent contrast enhancement of the APS (Figure 6) that was refurnished from an arterial vessel coming from the left gastric artery (Figure 7). A second angiography and subsequent embolization managed to stop APS refilling. Therefore, after two days a left hepatectomy was performed. Pathological examination revealed a trabecular HCC with satellitosis, vascular invasion and necrosis (pT3a, G3), a tumor thrombus in the left branch of the portal vein, and active cirrhosis in liver parenchyma. The postoperative course was complicated by mild signs of liver failure with ascites (about 700 mL die) which resolved after treatment with diuretics and iv administration of albumin (Grade II according to Dindo-Clavien classification of complications following surgery) [5]. The patient was discharged in postoperative day (POD) 16. The patient is alive and disease free at 8 months after left hepatectomy.

## Discussion

HCC is associated with the presence of intralesional APS regarding the common portal vein in approximately 60% [6], or the main portal vein branches in 30% of patients according to data reported by Okuda et al [7].

The presence of a high flow APS with hemodynamically significant consequences, results in the creation or worsening of portal hypertension, with potentially life threatening complications such as oesophageal varices rupture, refractory ascites, and liver encephalopaty [1-3].

The diagnosis of APS is reached through the execution of a contrast enhanced abdomen CT, revealing the absence of atypical HCC radiologic features, a clear enhancement of the portal vein axis contemporary to the hepatic artery during the arterial phase of the examination and a lack of enhancement during the arterial phase, since the blood flow to the lesion is reduced because of the APS [8,9]. The diagnosis of multifocal HCC with APS makes two issues arising: first, the treatment of APS and, secondly, the treatment of HCC out of Barcelona criteria [10].

Treatment options range from medical therapy alone (Sorafenib), interventional radiology procedures (TAE to treat the APS and/or transarterial chemoembolization for the treatment of hepatic multifocal lesions) with or without medical therapy, to radiological treatment of the shunt (APS-TAE) and subsequent surgical liver resection [11-14].

In this case report, in decision-making process two sets of issues were taken into account: in the one hand, the issue of APS in patients with HCC and, in the other the treatment of hepatocellular malignancy out of Barcelona criteria.

Regarding the first point, the emergency treatment of acute bleeding from esophageal varices may be made by endoscopic ligation or sclerotherapy, and finally by TIPS or surgical shunt if the control of portal hypertension is not effective [15].

It is remarkable that in patients with APS the control of portal hypertension is consequent to the treatment of the shunt itself. Even if the rates of recanalization are high, as reported in the literature [16], in this case it was decided to proceed with embolization of arterial blood vessels feeding the shunt, with the dual aim of reducing the risk of portal hypertension related complications (also aggravated by portal vein thrombosis) and of gaining better hemostatic control intraoperatively.

The need for a double session of embolization was established by radiological finding of shunt persistence due to arterial vessels originating from the left gastric artery.

Control of portal hypertension resulted in ascites resolution and re-classification of the patient in class A5 according to Child Pugh: if not, the patient would have taken two points for ascites resulting in B7. This means that transient complications of portal hypertension tend to overestimate patient's class of risk, especially regarding postoperative liver failure. After shunt treatment indeed, the patient was reported to the appropriate class of Child and during the postoperative course, the patient developed only mild and transient signs of liver failure. Ishii et al. noted that in the presence of APS the green indocyanin retention test proved to be unreliable [17], since it overstates functional parenchymal impairment [13].

Timing for surgery has to be decided in a case by case analysis, considering the balance between complete acute complication resolution and the risk of ischemic or septic complications and APS recanalization.

A proper evaluation of the hepatic functional reserve, prevents inappropriate exclusion of patients from the only potentially curative treatment, because of risk overestimation.

In the present case report, the patient presented with multifocal disease, portal hypertension and malignant PVT: the long term prognosis of these patients is quite poor, so that Barcelona criteria do not indicate surgery as the treatment of choice, since the 5-year survival rate is less than 50% [10,18]. Despite of this, the most experienced surgical centers continue to perform liver resection out of Barcelona indications [19], suggesting that good long-term results are achievable, better than those reported after non-curative treatments [10].

In particular, patients with PVT have a median survival of 2.7 months without treatment [20]. On the contrary, following surgery, several authors report 3 years survival rates ranging between 0 and 43% depending on the series [21-24]. Furthermore, the presence of portal hypertension is not a contraindication itself to invasive approach or a predictor of outcome, as suggested by Capussotti [25] and other authors [26,27].

## Conclusion

In conclusion, the diagnostic and therapeutic strategy proposed in this case is aimed to both the resolution and prevention of acute complications of portal hypertension and to offer a chance of long term survival thanks to oncologic treatment. Transient complications of portal hypertension have to be corrected before deciding therapeutic strategy, as they tend to overestimate patient's class of risk and may induce inappropriate exclusion of patients from surgery that is actually able to improve long term survival.

## Consent

Written informed consent was obtained from the patient for publication of this Case report and any accompanying images. A copy of the written consent is available for review by the Editor in Chief of this journal.

## List of abbreviations

HCC: Hepatocellular carcinoma; APS: Arterioportal shunt; HCC: Hepatocellular carcinoma; PVT: Portal vein thrombosis; TAE: Trans arterial embolization; POD: Post operative day.

## Competing interests

The authors declare that they have no competing interests.

## Authors' contributions

FR had the idea of the case report and wrote the manuscript. FC collected all patients data, follow up and radiological images and participated to manuscript preparation. MP and GF provided technical assistance and supported in discussion elaboration to magnify case report importance. LA contributed to discussion writing, performed the surgical operation and elaborated patient's therapeutic strategy.

All authors have read and approved the final manuscript.
